# Rejection of large HPV-16 expressing tumors in aged mice by a single immunization of VacciMax^® ^encapsulated CTL/T helper peptides

**DOI:** 10.1186/1479-5876-5-26

**Published:** 2007-06-07

**Authors:** Pirouz M Daftarian, Marc Mansour, Bill Pohajdak, Antar Fuentes-Ortega, Ella Korets-Smith, Lisa MacDonald, Genevieve Weir, Robert G Brown, W Martin Kast

**Affiliations:** 1 ImmunoVaccine Technologies Inc., Halifax, NS, B3J 3R1, Canada; 2 Department of Microbiology & Immunology, the University of Miami, Miami, FL, USA; 3 Dept. of Molecular Microbiology & Immunology and Norris Comprehensive Cancer Center, University of Southern California, Los Angeles, CA, USA

## Abstract

The incidence of cancer increases significantly in later life, yet few pre-clinical studies of cancer immunotherapy use mice of advanced age. A novel vaccine delivery platform (VacciMax^®^,VM) is described that encapsulates antigens and adjuvants in multilamellar liposomes in a water-in-oil emulsion. The therapeutic potential of VM-based vaccines administered as a single dose was tested in HLA-A2 transgenic mice of advanced age (48–58 weeks old) bearing large palpable TC1/A2 tumors. The VM-based vaccines contained one or more peptides having human CTL epitopes derived from HPV 16 E6 and E7. VM formulations contained a single peptide, a mixture of four peptides or the same four peptides linked together in a single long peptide. All VM formulations contained PADRE and CpG as adjuvants and ISA51 as the hydrophobic component of the water-in-oil emulsion. VM-formulated vaccines containing the four peptides as a mixture or linked together in one long peptide eradicated 19-day old established tumors within 21 days of immunization. Peptide-specific cytotoxic cellular responses were confirmed by ELISPOT and intracellular staining for IFN-γ producing CD8^+ ^T cells. Mice rendered tumor-free by vaccination were re-challenged in the opposite flank with 10 million HLF-16 tumor cells, another HLA-A2/E6/E7 expressing tumor cell line. None of these mice developed tumors following the re-challenge. In summary, this report describes a VM-formulated therapeutic vaccine with the following unprecedented outcome: a) eradication of large tumors (> 700 mm^3^) b) in mice of advanced age c) in less than three weeks post-immunization d) following a single vaccination.

## Background

Decreased immunity in the elderly has been documented and associated with decreased T cell differentiation and aging of naïve T cells, as well as aging of peripheral memory cells [1-3]. To counter decreasing immunity in the elderly, studies on influenza vaccination have shown that immune responses in the elderly could be elevated by designing vaccines with enhanced immunogenicity, by using strong adjuvants and increasing access to professional antigen presenting cells. This resulted in stimulating the Th1 arm of the immune system, and increasing durable memory T cells [4,5]. We have previously described such a highly effective single administration vaccine platform (VacciMax^®^,VM) that uses liposomes in a water-in-oil emulsion to deliver a peptide derived from HPV 16 E7 having a CTL epitope fused to PADRE with CpG adjuvant [6]. The efficacy of this vaccine platform was evaluated using a well-described mouse model based on HPV 16-expressing C3 tumor cells [7]. The present study used VM in a different tumor model, namely a TC1/A2 cell line that expresses human class I MHC and HPV16 in HLA A2-mice [8,9]. The aim of this study was to investigate whether VM-formulated vaccine incorporating human MHC restricted HPV-16 peptides would be efficacious in rejection of large established tumors in humanized mice of advanced age.

## Methods

### Mice and cell lines

Pathogen-free 8–12 week old HLA-A2 transgenic breeding mice, HLA-A*0201 (C57BL/6-Tg, HLA-A2.1, 1Enge/J, Jackson Laboratories) were housed under filter top conditions with water and food *ad libitum*. Institutional animal care and use guidelines were followed for all experiments. The mice were maintained until age 48–58 weeks. The transgenic HLA-A2 mice express the α1, α2 and α3 domains of HLA-A2.1.

TC1/A2 tumor cells were primary lung epithelial cells co-transformed with HPV-16 E6, HPV-16 E7, and *ras *oncogenes (8). The TC1/A2 tumor cell line was cultured in RPMI 1640 (Sigma, St. Louis, MO) supplemented with 10 % heat-inactivated fetal calf serum (Hyclone), 2 mM L-glutamine (Gibco), 5 mM 2-mercaptoethanol (Gibco), penicillin and streptomycin (100 μg/ml). Cells were incubated at 37°C/5% CO_2_.

The HLF16 tumor cell line was established from heart and lung tissue from HLA-A2 D^d ^mice. HLF16 tumor cells expresses E6, E7 with a deletion of (E7:_49–57, RAHYNIVTF). HLF16 tumor cells do not present this very immunogenic H-2D^b ^epitope therefore, rejection of the tumor would most likely be mediated by immune responses against the HLA-A*0201-restricted peptides (13).

### Peptides and oligonucleotides (ODN)

The HLA A*0201 restricted HPV 16 peptides, which have been previously described [10], were purchased from Dalton Chemical Laboratories Inc. (Toronto, ON, Canada). The peptide sequences are derived from the E6 and E7 proteins of type 16 HPV and are as follows: (E7:11–20; YMLDLQPETT), (E7: 82–90; LLMGTLGIV), (E7:86–93; TLGIVCPI) and (E6:29–38; TIHDIILECV). The peptides were incorporated in the vaccine as a mixture (25 μg of each peptide/dose), or as a single fusion protein in which the 4 peptides are joined together with AAY spacers for optimal proteosomal cleavage [11]. The resulting long peptide (hereafter designated AB2) (TIHDIILECVAAYYMLDLQPETTAAYLLMGTLGIVAAYTLGIVCPI) was administered at 100 μg/dose. All vaccine formulations contained PADRE (AKXVAAWTLKAAA-OH; 25 μg/dose) and species specific CpG ODN 1826 (5'-TCCATGACGTTCCTGACGTT-3', Coley Pharmaceutical, Wellesley, MA) at 50 μg/dose.

### Tumor challenge

Mice were challenged with TC1/A2 tumor cells (10^5 ^cells/mouse) implanted subcutaneously in the left flank. Tumor size was measured every 5 days and is reported as the average tumor size in five mice or as tumor size in individual mice. Sixty days post-TC1/A2 implantation, tumor free mice were re-challenged in the opposite flank by 10^7 ^HLF16, a heart and lung fibroblast tumor cell line transfected with E6, E7 that lacks the H-2 ^b^restricted HPV 16 E7 49–57 epitope forcing all the T cell responses to recognise these tumor cells only through HLA-A*0201 restriction [12,13].

### Therapeutic immunization

Nineteen days post-TC1/A2-challenge, mice (5 mice/group) received a single subcutaneous (s.c.) injection of vaccine containing either the four peptides, the AB2 peptide or E7:82–90 alone. The HPV E6/E7 peptides were encapsulated in VM as previously described [6]. In brief, lecithin and cholesterol in a ratio of 10:1 (0.2 g lecithin and 0.02 g cholesterol/dose) were dissolved in chloroform/methanol (1:1; v/v) and the solution was filter-sterilized using a PTFE 0.2 μm filter. Chloroform and methanol were removed under reduced pressure using a rotary evaporator and traces of the solvents were removed from the resulting thin lipid layer *in vacuo*. For liposome encapsulation, peptides with CpG and PADRE were dissolved in sterile PBS and the resulting solution added to the thin lipid layer with mixing to form liposomes. The resulting suspension of liposomes was emulsified in Montanide ISA51 (Seppic, France) by adding the liposome/PBS suspension to ISA51 to form a water-in-oil emulsion (PBS:ISA51; 1:1,v/v; 100 μl/dose). Control mice were injected with vaccine that contained all components of the test vaccines except liposomes, CpG ODN alone or phosphate buffered saline (PBS, 100 μL/injection).

### *Ex vivo *analysis of antigen-specific T cells by ELISPOT

Activated antigen-specific cytotoxic T-cells in splenocytes harvested from immunized C57BL/6 mice were detected using the BD ELISPOT kit following the instruction manual (BD Bioscience, San Diego, CA). Briefly, on day 8 post-immunization, a 96-well nitrocellulose plate was coated with capture antibody, a purified anti-mouse IFN-γ antibody, by incubation overnight at 4°C, then blocked with complete media. Splenocytes were added to wells at an initial concentration of 10^6 ^cells/well in a volume of 100 μl and a row of serial dilutions prepared. Cells in a dilution series were either unstimulated or stimulated with relevant or irrelevant peptides (10 μg/ml).

PMA (5 ng/ml, Sigma) and ionomycin (500 ng/ml, Sigma), served as positive controls and irrelevant peptide and media alone served as negative controls. The plate was incubated overnight at 37°C/5% CO_2_. Next day, the plate was incubated with detection antibody (a biotinylated anti-mouse IFN-γ antibody), for 2 hours at room temperature. Unbound detection antibody was removed by washing and the enzyme conjugate (Streptavidin-HRP) was added. Following 1 hour incubation at room temperature, unbound enzyme conjugate was removed by washing and the plate was stained with an AEC substrate solution for 20 minutes. The plate was washed, allowed to air dry overnight, and foci of staining were counted using a magnifying lens.

### Intracellular cytokine staining

Spleens from mice were removed and splenocytes harvested as previously described. The splenocytes were washed twice with complete RPMI (500 × g, 5 minutes) then suspended in RPMI (10^7 ^cells/ml). Splenocytes (10^6^cells/well) were added to each well of a 96-well flat bottom plate and incubated with the irrelevant peptide or HPV 16 E7 peptides at a final concentration of 3 μg/ml. Duplicates were prepared for each peptide, then plates were incubated for 6 hours at 37°C/5 % CO_2_.

Intracellular cytokine staining used the procedure described by the manufacturer (Cytofix Cytoperm™ kit, BD Biosciences, Mississauga, ON). In brief, 2 hours after addition of stimulants, Golgistop (1 μl) was added to each well and the plates were incubated (37°C/5% CO_2_) for an additional 4 hours. Cells were washed twice with staining buffer (200 μl, 0.01% sodium azide and 1% bovine serum albumin or 1% fetal calf serum in PBS). Cells were incubated (20 minutes, 4°C, in the dark) with anti-CD8 FITC conjugated antibody (1 μl), washed with staining buffer and incubated (30 minutes at 4°C in the dark) with Cytofix (50 μl). Finally cells were incubated with APC-labeled anti-IFN-γ antibody (1 μl; 30 minutes at 4°C in the dark). Unbound antibody was removed by washing, cells were resuspended in perm/wash buffer and transferred to FACS tubes. Staining was assessed by FACSCalibur (BD Biosciences, San Jose, CA) and analyzed using CellQuest software.

### Statistical analysis

Data are expressed as the mean ± SD and statistical significance was determined using paired two-tailed Student t-test (p < 0.05).

## Results

### Eradication of large TC1/A2 tumors

HLA-A2 transgenic mice represent a versatile animal model for preclinical studies of HLA-A*0201 restricted CTL responses and represent a mouse model which is closer than C57BL6 for human cervical cancer. One limitation of H-2-restricted studies is antigen processing, therefore, human MHC class I epitopes can not be tested in this system. HLA transgenic mice, however, may serve as a bridge between the mouse and human MHC restricted responses. Independent reports have demonstrated that HLA-A*0201 transgenic mice recognize CTL epitopes detected by human CD8+ T cells that express the same MHC [14,15]. Straight A2 mice are more likely to mount high affinity CTL responses against peptides since the mouse CD8 molecules have a low affinity for human HLA α3 domains. Such weak binding suggest that an effective interaction between CTLs and tumor cells requires an overall high avidity which is likely to be provided by high affinity CTLs.

A major limitation of preclinical tumor immunotherapy studies is their failure to reject well-established tumors resulting in a very modest success rate when such studies move into clinical trials. The microenvironment of early stage tumors (3, 4, 5, or 6-day old) is different than the microenvironment of advanced stage tumors which may be more representative of human cancer. Here, we examined the ability of VM-formulated vaccines to reject 19-day old tumors of large sizes.

**Figure 1:**
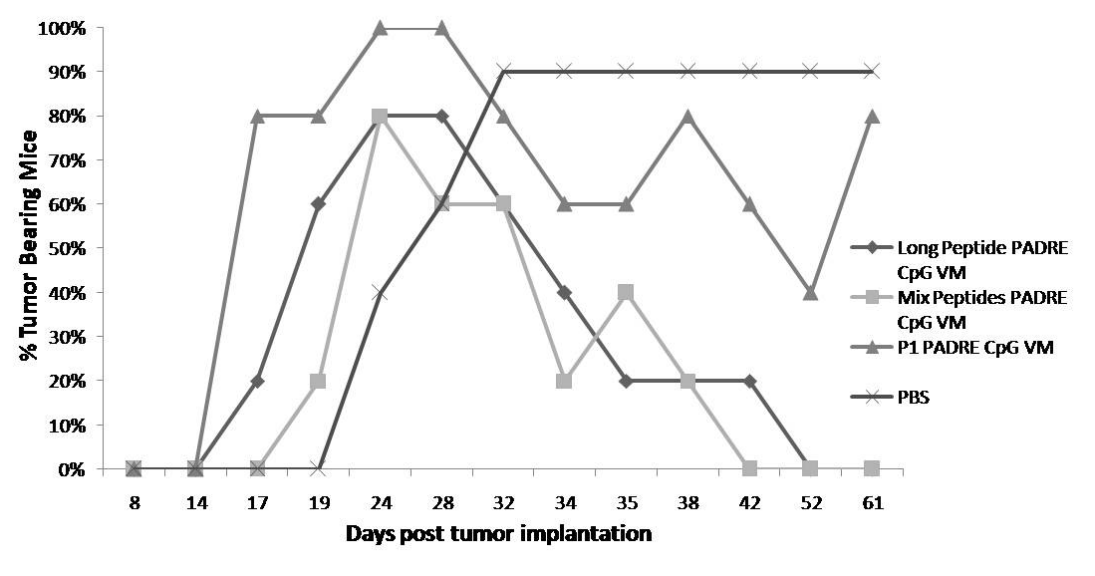
Immunization of mice against the four peptide mixture derived from HPV 16 E6/E7 proteins of type 16 HPV (squares) or the same peptides joined together in a long peptide (diamonds) eradicated tumors from all treated mice. Immunization of mice against a single peptide E7: 82–90 eradicated TC1/A2 tumors from 60% of treated mice (triangles). In contrast, all mice that received PBS developed tumors (crosses). These results suggest that immunization against more than one peptide antigen results in increased eradication of tumors and that multiple peptide antigens can be administered as a mixture or fused into one peptide.

To evaluate the therapeutic effect of vaccination against peptide antigens delivered with VM, HLA-A2 mice were implanted with TC1/A2 tumor cells that cause the establishment of large tumors by day 19 post-implantation. Mice in four treatment groups received: 1) PBS, 2) E7: 82–90 , 3) the mixture of four HPV E6/E7-derived peptides (E7:11–20; E7: 82–90; E7: 86–93; E6: 29–38) that contain human CTL epitopes or 4) the same four peptides fused together in a long peptide (AB2). All mice injected with PBS developed tumors (Fig. 1). Forty percent of mice vaccinated against E7: 82–90 carried tumors up to 60 days post-tumor implanation when the trial was terminated. In contrast, all mice treated with a mixture of the four peptides or the four peptides fused together were tumor-free by day 60 post-tumor implantation.

**Figure 2:**
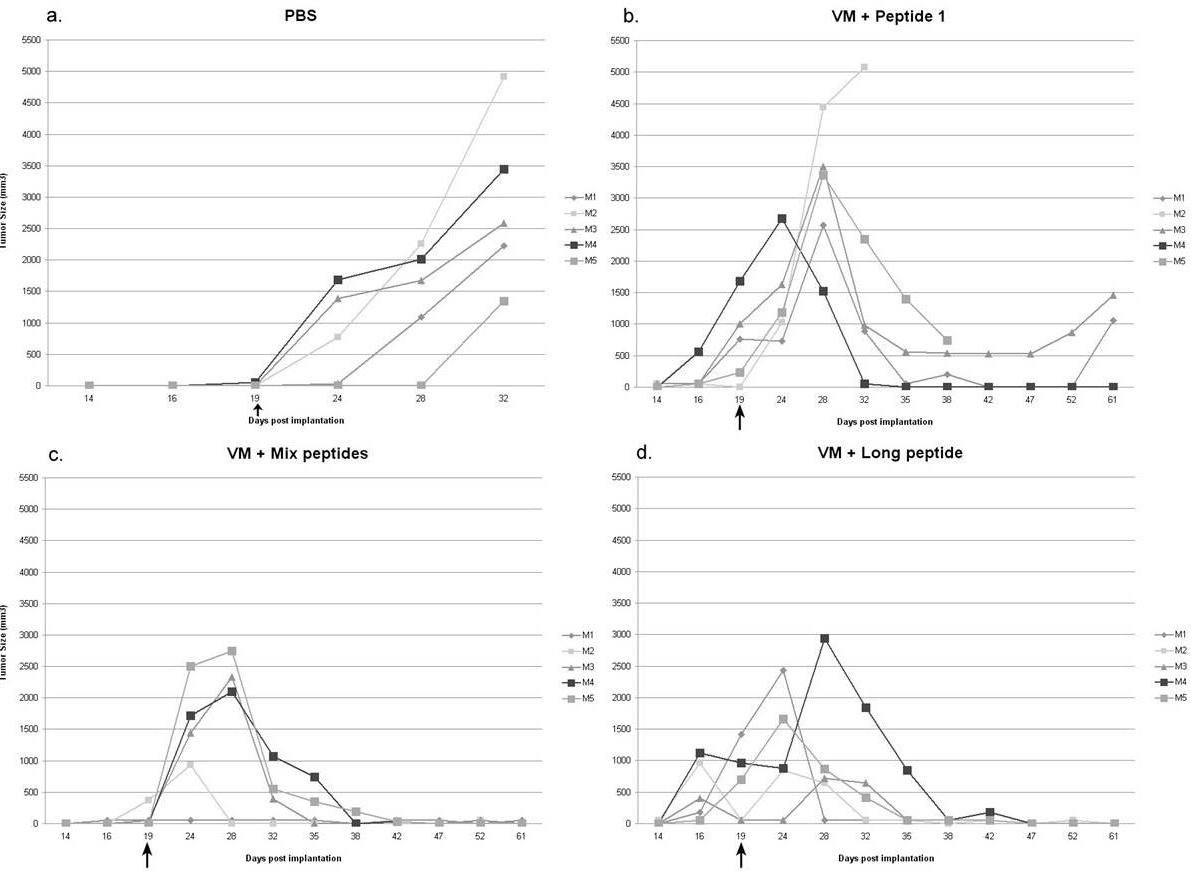
Development of TC1/A2 tumors in five mice treated with PBS (panel A), E7: 82–90 (panel B), peptide mixture (panel C) or fused peptide (AB2; panel D). The arrows indicate time of vaccination.

**Figure 3:**
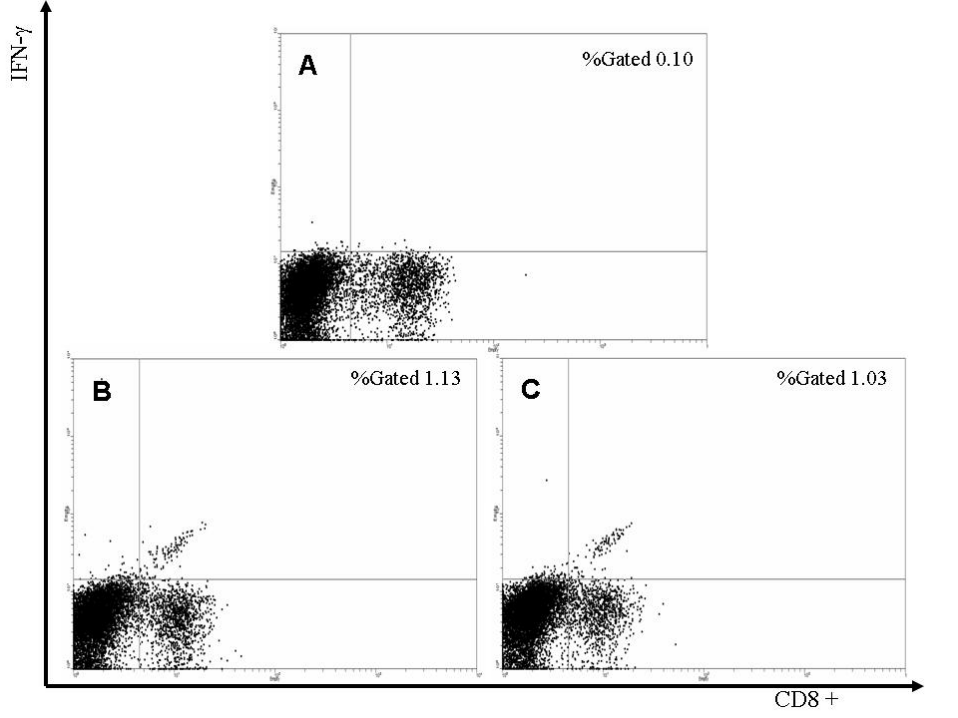
*Ex vivo *intracellular IFN-γ staining of splenocytes from mice immunized against E7: 82–90 alone or a mixture of four peptides consisting of E7: 11–20, E7: 82–92, E7: 86–93 and E6: 29–38. Splenocytes not stimulated with exogenous peptide produced background levels of IFN-γ staining splenocytes (panel A). Only immunization against E7: 82–90 (panel B) or a mixture of the four peptides (panel C) caused expansion of CD8 ^+^/IFN-γ ^+ ^T-cells above background levels.

**Figure 4:**
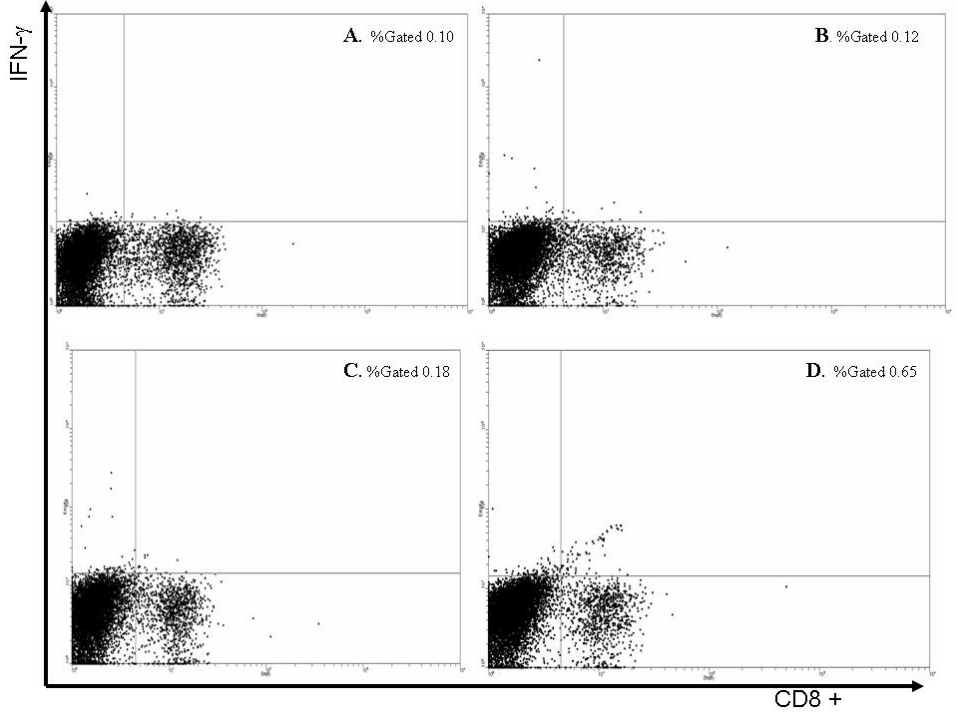
*Ex vivo *intracellular IFN-γ staining of splenocytes from unimmunized mice or mice immunized against E7: 11–20, E7: 86–93 or E6: 29–38 alone. Splenocytes not stimulated with exogenous peptide produced background levels of IFN-γ staining splenocytes (panel A). as did splenocytes from immunized with E6: 29–38 (panel B) and E7: 86–93 (panel C) Only immunization against E7: 11–20 (panel D) caused expansion of CD8 ^+^/IFN-γ ^+^T-cells above background levels.

Measurement of tumor size in individual mice indicated that tumors were eradicated at different rates in individual mice (Fig. 2). Tumor growth was similar in all mice in the control group that received PBS (Fig, 2A). Of the four peptides in the mixture, E7: 82–90 was selected as having the most potential to produce specific CTL responses based on intracellular cytokine staining and ELISPOT results (see Fig. 3 &4). Immunization against E7: 82–90 reduced tumor size but only eradicated the tumor of one mouse by day 40 post-tumor implantation (Fig. 2B). Tumors that were reduced in size in the other four mice, increased in size after day 40. Immunization with the peptide mixture caused tumor eradication by day 25 in one mouse, by day 30 in another mouse and in the remaining 3 mice by day 40 (Fig. 2C). Similarly, immunization with the long peptide caused tumor eradication by day 25 in one mouse, day 35 in another mouse and in the remaining 3 mice by day 40 (Fig. 2D). The tumor in one mouse from the latter group increased in size to 5500 mm^3 ^before reduction in tumor size was observed.

### Intracellular cytokine staining

To measure the relative ability of each peptide in the four peptide mixture to induce a CTL response, mice were immunized by a single administration of each peptide in the mixture alone or a mixture of the four peptides in VM. Fourteen days post-immunization, splenocytes were cultured for 6 h in the presence of each peptide, the peptide mixture (in accordance with the immunization regime) or without added peptide. *Ex vivo *intracellular IFN-γ staining of splenocytes from mice immunized against E7: 82–90 demonstrated that the proportion of IFN-γ positive CD8^+ ^T-cells was higher (1.13% of splenocytes) when splenocytes were exposed to E7: 82–90 compared to no exposure to exogenous peptide (0.10% of splenocytes; Fig. 3). The proportion of IFN-γ positive CD8 ^+ ^T-cells in splenocytes from mice immunized against the peptide mixture and stimulated with the peptide mixture was similar (1.03% of splenocytes). The proportion of IFN-γ positive CD8 ^+ ^cells in splenocytes from mice immunized against E7: 11–20, E7: 86–93 or E6: 29–38 and stimulated with homologous peptide were 0.65, 0.18 and 0.12 % of splenocytes respectively (Fig. 4). The results indicate that E7: 82–90 peptide induced the strongest CTL response.

## Discussion

Malignancies pose a burden on health worldwide but in particular for the elderly. High morbidity and mortality are attributable to limited current therapies, therefore, there is a need for a new generation of vaccines that are cost effective, safer and yet mount robust, effective and durable immune responses [16]. While in general, vaccines are beneficial for the elderly, there is a need to improve vaccine efficacy for people of advanced age [4,5]. Previously, we reported that a single vaccination of a HPV 16 E7-derived peptide coupled to PADRE in VM induces immune responses that are efficacious, potent, safe and durable [6]. To extend these results to a more clinically appropriate HPV cancer model, the TC1/A2 and HLF 16 tumor cell lines in aged HLA-A2 transgenic mice were chosen. This model requires the use of peptide antigens that are presented by HLA-A*0201 and serves as a bridge between pre-clinical and phase I clinical trials. Previous studies that used the TC1/A2 or HLF-16 in this humanized mice model reported a prophylactic or prophylactic/therapeutic benefit from vaccination respectively suggesting that cancer immunotherapy offers promise [9,12,13]. However, this is the first report demonstrating rejection of large established 19-day old TC1/A2 tumors in mice of advanced age. The results reported in this study indicate that effective immunotherapy against human cervical cancer is possible. In particular, this study used VM to administer four peptides as a mixture or as a single peptide formed by coupling the four peptides together. Both treatments resulted in eradication of tumors from all treated mice. Furthermore, none of these mice developed tumors when re-challenged with 10^7 ^HLF-16 tumor cells, which is a different HLA-A2/E6/E7 expressing tumor cell line. This suggests that the cytotoxic immune response generated with VM was robust enough to prevent regeneration of any of the potentially eradicated tumors.

The antigens employed in this report are an improvement over the antigen composition previously reported [6]. Hitherto, the antigen was chemically coupled to PADRE as a means of keeping antigen and adjuvant together. The liposomes in VM have the potential advantage of keeping all vaccine ingredients together by encapsulation. Eradication of tumors by vaccines described in this report suggests that chemical coupling of antigen to PADRE is not essential for a successful cancer vaccine and that encapsulation of vaccine ingredients in VM is sufficient to stimulate PADRE-induced T cell help for the expansion of CTL responses. Likewise, chemical coupling of peptide antigens is unnecessary for an effective multiple-peptide based vaccine provided VM is used in vaccine delivery.

In summary, the VM vaccine platform containing peptide antigens in two different formats, namely a mixture of single peptides or the same peptides linked together in a single long peptide induces a robust CTL response. The immune responses induced by each peptide antigen format were shown to be highly efficacious using a cancer model based on humanized mice of advanced age.
